# Colloid Carcinoma of the Pancreas: Case Report and Review of the Literature

**DOI:** 10.1089/crpc.2016.0006

**Published:** 2016-06-01

**Authors:** Sonia T. Orcutt, Domenico Coppola, Pamela J. Hodul

**Affiliations:** ^1^ Department of Gastrointestinal Oncology, H. Lee Moffitt Cancer Center & Research Institute, Tampa, Florida.; ^2^ Department of Anatomic Pathology, H. Lee Moffitt Cancer Center & Research Institute, Tampa, Florida.

**Keywords:** colloid carcinomas, IPMN, pancreatic cancer

## Abstract

**Background:** Colloid carcinoma of the pancreas is a rare type of pancreatic cancer that has a more indolent course and superior long-term survival compared to ductal adenocarcinoma. There is a dearth of literature describing this diagnosis due to its rarity and its only recent recognition as a distinct clinical entity. We present two cases of patients with colloid carcinoma and discuss the presentation and management of this disease.

**Case Presentation:** A 58-year-old man with repeated bouts of pancreatitis and a 72-year-old woman with symptoms of pancreatic exocrine and endocrine insufficiency were both found to have cystic masses in the head of the pancreas. Both were identified as having at least mixed main duct/side branch intraductal papillary mucinous neoplasms (IPMNs) on appropriate workup with additional imaging and endoscopy. Pancreaticoduodenectomy was recommended. Both patients, however, were noted to have high-grade dysplasia at the resection margin intraoperatively on frozen section, and thus, total pancreatectomies were performed. Final pathology in each case demonstrated colloid carcinoma with no nodal spread of disease. The patients recovered well. Adjuvant chemotherapy was recommended.

**Conclusion:** Colloid carcinoma of the pancreas is a rare pathologic diagnosis and is frequently associated with IPMN. Colloid carcinomas tend to present at earlier stages than do ductal adenocarcinomas and are known to have improved long-term survival. Surgical and systemic options for treatment parallel that of ductal adenocarcinoma due to the rarity of the diagnosis and the lack of trials assessing therapy for this specific diagnosis.

## Introduction

Colloid carcinoma of the pancreas, also known as mucinous noncystic carcinoma, is a rare pancreatic neoplasm and represents about 1% of all pancreatic tumors.^1,2^ As it is uncommonly diagnosed, there is a dearth of information in the literature about this diagnosis. Over the last two decades, there has been increased recognition of colloid carcinoma as a distinct clinical entity, as it was frequently previously categorized as ductal adenocarcinoma or misdiagnosed as mucinous cystadenocarcinoma or signet-ring cell carcinoma of the pancreas.^2^

Colloid carcinomas most often develop in the head of the pancreas, frequently associated with intraductal papillary mucinous neoplasms (IPMNs), or less commonly mucinous cystic neoplasms.^2^ Histologically, colloid carcinoma of the pancreas is characterized by an extensive amount of mucin, with neoplastic cells floating within large mucin pools.^1^ Descriptions of these tumors have also included irregular contours of the mucin lakes and tumor cells typically being cuboidal or forming stellate clusters centrally within the mucin, but occasionally resembling signet-ring cells.^2^

The importance of colloid carcinoma as a distinct clinical entity lies in its superior long-term prognosis compared to typical pancreatic ductal adenocarcinoma, with reports of 5-year survival 40–60% versus 10–15%, respectively.^3,4^ We present two cases of patients with final pathologic diagnoses of colloid carcinoma. Both patients, due to associated IPMN, required total pancreatectomies as surgical treatment. We also discuss the presentation, management, and prognosis of this pathologic diagnosis.

## Presentation of Cases

The first patient is a 58-year-old man who was a previous smoker who presented to an outside hospital with abdominal pain and was diagnosed with gallstone pancreatitis. However, despite having a laparoscopic cholecystectomy, he continued to have episodes of pancreatitis yearly for the next 3 years. Computed tomography (CT) scan demonstrated a pancreatic duct dilated throughout its course to a maximum size of 8 mm without a discrete pancreatic mass in the head of the pancreas. Endoscopic retrograde cholangiopancreatography confirmed a dilated pancreatic duct with a suspected filling defect of the common bile duct, after which the patient underwent sphincterotomy and stent placement.

**FIG. 1.**
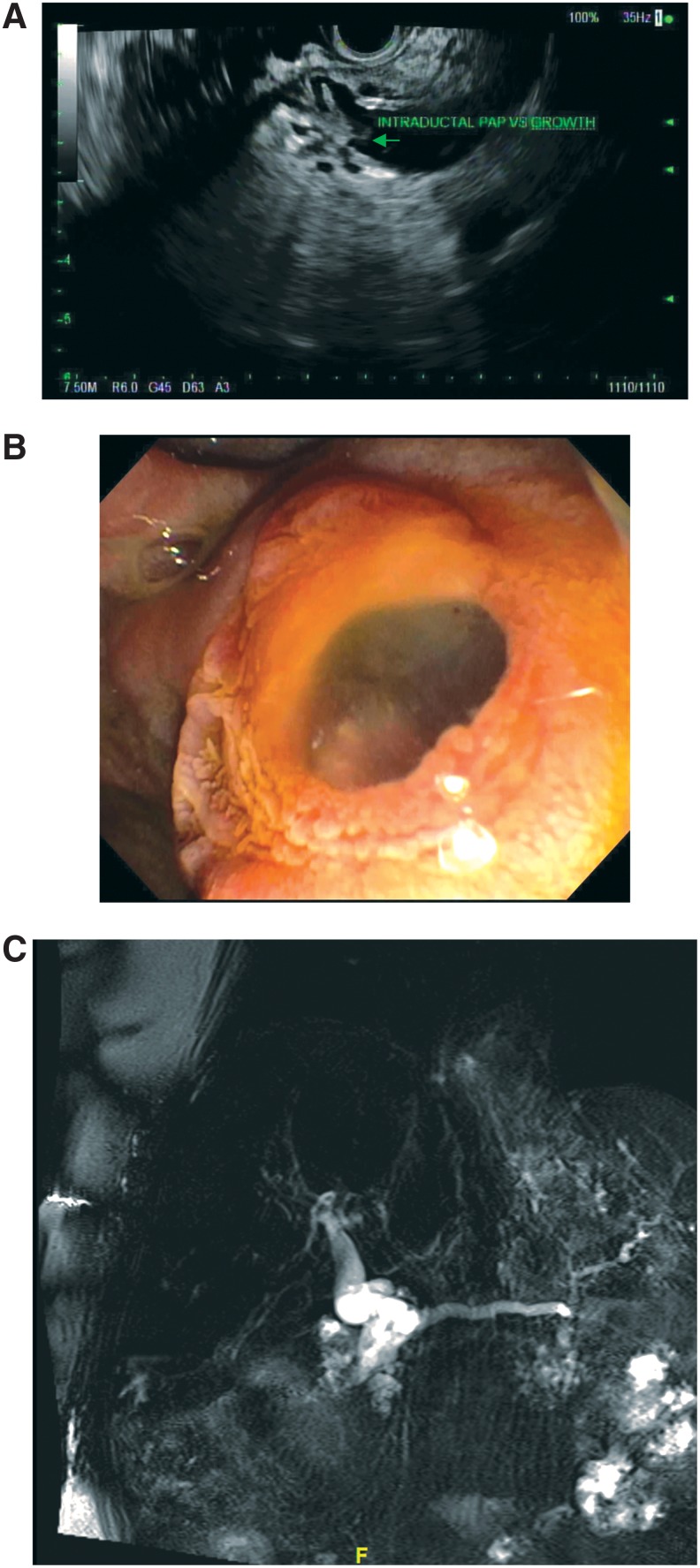
**(A)** Endoscopic ultrasound demonstrating a papillary growth in a dilated pancreatic duct in the head of the pancreas of the first patient. **(B)** Endoscopic image of the first patient's major duodenal papilla with mucin extrusion, suggestive of IPMN. **(C)** Magnetic resonance cholangiopancreatography demonstrating the second patient's diffuse pancreatic dilatation. IPMN, intraductal papillary mucinous neoplasm.

He then presented to our institution for consultation. Further workup with endoscopic ultrasound (EUS) was performed, which also confirmed the dilated pancreatic duct, but noted irregular contour of the duct. In addition, free mucin was seen exiting the main papilla, and there was a papillary growth noted in the pancreatic duct in the head of the pancreas, all of which was consistent with a main duct IPMN (Fig. 1). Preoperative laboratory values, including CA19-9, were within normal limits. Of note, it was unclear if the dilatation of the pancreatic duct was secondary to obstruction from the mass in the pancreatic head or due to main duct IPMN involving the entirety of the duct.

He was scheduled for surgical resection after the stated workup. He was planned for a Whipple procedure (pancreaticoduodenectomy) with possible total pancreatectomy if intraoperatively the main duct was found to be involved by IPMN with high-grade dysplasia. Transection of the pancreatic neck identified cells of high-grade dysplasia free floating near the margin (although exclusive of the margin). In addition, upon probing the pancreatic duct in the tail of the pancreas, the probe did not slide smoothly, suggestive of additional growths in the duct. Due to his young age and high risk of developing pancreatic cancer, a completion pancreatectomy and splenectomy were performed. He was reconstructed in a Roux-en-Y manner. He recovered well from surgery without any postoperative complications.

His final pathology demonstrated a 1.4 cm moderately differentiated colloid carcinoma arising within main duct IPMN (intestinal type) with high-grade dysplasia in addition to multiple foci of pancreatic intraepithelial neoplasia. Staining of the tumor was strongly positive for MUC2 and weakly positive for MUC1 and MUC5. The tumor involved the peripancreatic soft tissue, but all margins were negative. 0 of 39 lymph nodes was involved with tumor. His final pathologic staging was pT3N0Mx. He was, therefore, referred for medical oncology consultation. He was recommended for adjuvant chemoradiation with gemcitabine before and after fluorouracil-based chemoradiation,^5^ which he is currently receiving and tolerating well.

The second patient is a 72-year-old woman with a past medical history including hypertension, type II diabetes, and obesity, who presented with weight loss and steatorrhea. As her symptoms were suggestive of pancreatic exocrine insufficiency, and with the associated weight loss, she underwent a CT scan for additional evaluation. This demonstrated a suspicious 2.5-cm complex cystic pancreatic head mass with associated pancreatic gland atrophy and a dilated pancreatic duct. She was also noted to have fatty infiltration of the liver (although not overt cirrhosis) and ascites.

**FIG. 2.**
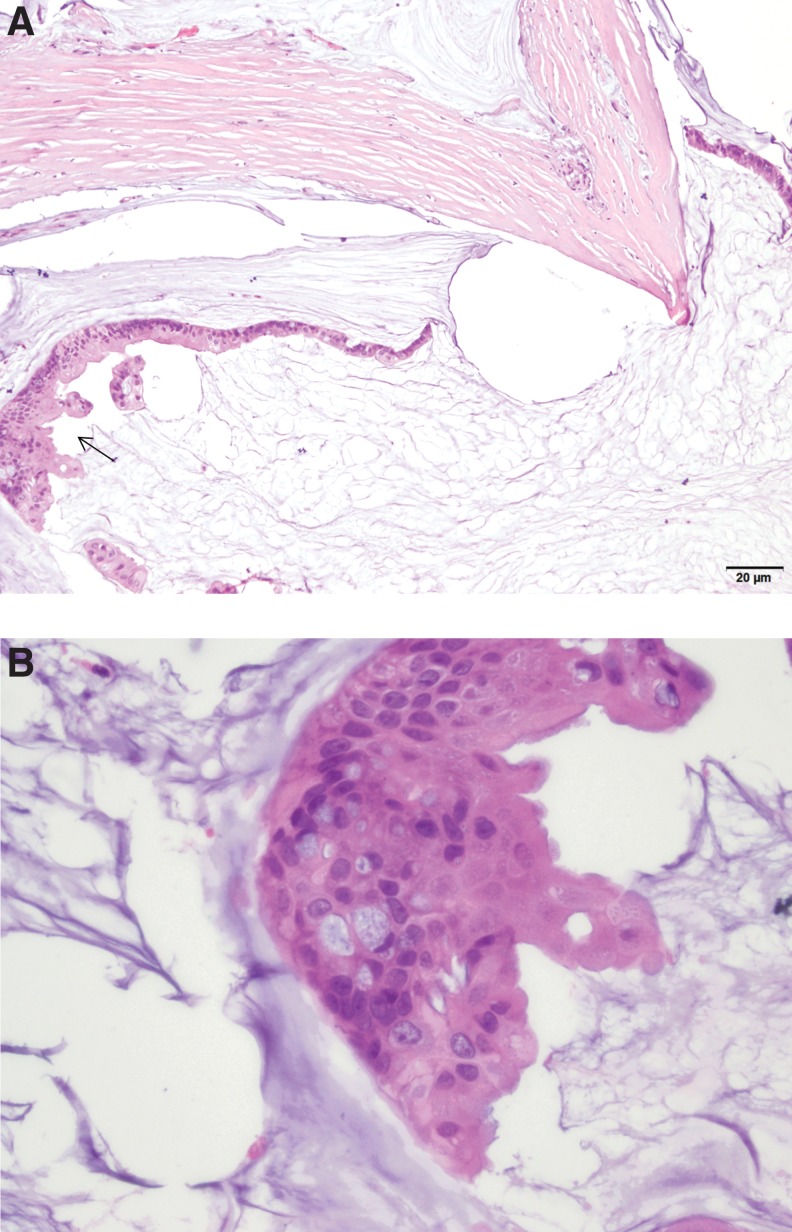
**(A)** Low-power pathologic slide from the second patient demonstrating the key features of colloid carcinoma, with extensive mucin pools and scattered neoplastic cells within them in the bottom of the photograph. Associated IPMN can also be seen as the ductal cells with papillary projections (arrow). **(B)** Higher power image depicting additional detail.

She was then referred for further evaluation. Magnetic resonance imaging (MRI) and magnetic resonance cholangiopancreatography demonstrated diffuse dilation of the pancreatic duct up to 7.5 mm with an associated cystic mass in the head of the pancreas, suggestive of main duct IPMN (Fig. 2). EUS was attempted, but was unable to be completed due to severe tortuosity of her esophagus. Positron emission tomography (PET) demonstrated heterogeneous metabolic activity within the pancreatic uncinate process and to a lesser degree within the body and tail. Additional workup included a paracentesis to evaluate the ascites, which was unremarkable and ultimately deemed secondary to poor nutritional status from her exocrine insufficiency. Laboratory results were within normal limits with the exception of tumor markers and a mildly low albumin (3.4 gm/dL). Carcinoembryonic antigen was elevated at 6.0 ng/mL, and CA 19-9 was elevated at 46.7 U/mL. She was placed on pancrelipase, which resolved her symptoms of exocrine insufficiency and helped improve her nutrition.

Due to the concern for main duct IPMN, she was referred for surgical resection. Again, it was unclear if the diffuse dilation of the pancreatic duct was secondary to involvement by main duct IPMN or due to proximal pancreatic duct obstruction. The patient was planned for a Whipple procedure with possible total pancreatectomy if high-grade dysplasia was noted intraoperatively at the resection margin. Upon transection of the pancreatic neck, IPMN with focal high-grade dysplasia was indeed noted at the margin. Due to the abnormal preoperative PET scan, in addition to the high-grade dysplasia at the margin, a completion pancreatectomy was performed. Due to her poor nutritional status preoperatively, a gastrojejunal feeding tube was placed at the time of the operation. She had an unremarkable postoperative course and was discharged with feeding tube supplementation.

Her final pathology demonstrated a 1.1 cm well-differentiated mucinous colloid carcinoma arising in a background of IPMN (intestinal type) with foci of high-grade dysplasia and intermediate grade dysplasia throughout the remaining pancreatic duct (Fig. 2). Staining of the tumor was positive for MUC1, MUC2, and MUC5. All margins were negative. Zero of 24 lymph nodes was involved with tumor. Her final pathologic staging was pT2N0Mx. She was referred to medical oncology for discussion of adjuvant therapy and will be initiating gemcitabine adjuvant chemotherapy^6^ in the near future.

## Discussion and Literature Review

Pancreatic cancer is expected to surpass breast cancer as the third leading cause of cancer-related deaths in the United States,^7^ despite improvements in recent years in locoregional surgical and radiation techniques and systemic treatments. However, despite its dismal prognosis and high mortality rate, there are rare types of pancreatic cancer with superior prognoses, but that are not well described in the literature. This article describes two cases of colloid carcinoma of the pancreas, which carries with it a significantly improved prognosis compared to typical pancreatic ductal adenocarcinoma.

Colloid carcinomas represent about 1% of pancreatic cancers.^1,2^ They are described as a variant of pancreatic ductal adenocarcinoma and in the majority are associated with IPMN. IPMNs when invasive are separated into either colloid carcinoma (as described in this study, arising from intestinal-type IPMN, and representing one-quarter of all invasive IPMNs) or tubular carcinoma (arising from the pancreatobiliary type of IPMN, morphologically similar to ductal adenocarcinoma, and representing three-quarters of invasive IPMNs).^3,8^ Studies assessing the long-term outcomes after resection of IPMN-associated carcinomas have suggested improved survival compared to ductal adenocarcinoma. This differential in survival is attributed to more favorable biology in IPMN-associated carcinomas, with lower T stage, fewer lymph node metastasis, fewer cases of poor tumor differentiation, less frequent vascular and perineural invasion, and fewer rates of microscopic margin involvement in IPMN-associated carcinomas.^3,4,9^ However, upon closer investigation, it was noted that IPMN-associated carcinomas are not a homogenous identity. Colloid carcinomas demonstrate improved median overall survival (40–60%) compared with tubular carcinomas, which behave similarly to pancreatic ductal adenocarcinoma (5-year overall survival 10–20%).^3,4,9^

A potential reason for the significantly improved prognosis for patients with colloid carcinoma compared to ductal adenocarcinoma may be due to alterations at a cellular level. It is thought that colloid carcinoma cells exhibit an inverse polarization, in which the basal aspect of cells secretes mucin toward the interface between the cells and the stroma (instead of toward the lumen), which then separates the cell from the underlying stroma. The mucin surrounding the epithelial cells then acts as a barrier, preventing further spread by inhibiting invasion of neoplastic cells and allowing for expansive growth of the tumor instead.^4,10^ This theory is supported by the differential staining of surface glycoproteins in colloid carcinoma cells compared to ductal adenocarcinoma; ductal carcinomas express the surface glycoprotein MUC1 on the luminal aspect or throughout the cells, whereas colloid carcinomas express MUC1 on the basal surface.^10^ Expression of MUC2, in contrast, has been identified in colloid carcinomas of the pancreas, but is rarely found in ductal carcinomas.^10^ In addition, MUC2 has been demonstrated to have tumor suppressor activity, which may also contribute to the indolent nature of colloid carcinomas.^10^

The cases described herein have similar characteristics to those previously described. The colloid carcinomas identified were both associated with intestinal-type IPMN. Both tumors expressed MUC1 and MUC2. Both patients presented with an early T stage and with no nodal involvement. Both required total pancreatectomies due to the extensive nature of IPMN involving the entire main duct (as opposed to tumor extension), which is commonly seen with the resection of invasive IPMNs.^3^

Due to the rarity of the diagnosis, colloid carcinomas are typically diagnosed after surgical resection, rather than on preoperative workup. Clinical presentation of patients with IPMN-associated carcinomas and ductal adenocarcinoma has been reported to be similar,^3,11,12^ with presentation in about the seventh decade of life and with similar presenting symptoms of abdominal pain, jaundice, and weight loss. There may be a slight male predilection in colloid carcinoma.^3,11^ Recommended diagnostic workup, including imaging with CT or MRI and EUS, is the same for ductal adenocarcinoma as for IPMN-associated carcinomas.

In addition, due to its rarity, guidelines for the treatment of colloid carcinomas of the pancreas specifically do not exist. However, surgery is recommended as the mainstay of treatment, which may include pancreaticoduodenectomy, distal pancreatectomy, or total pancreatectomy. Although the long-term survival is better in colloid carcinoma compared to ductal adenocarcinoma, studies stratifying by stage have found that with increasing stage, long-term survival is similar with these diagnoses.^3,4^ Therefore, adjuvant therapy similar to pancreatic ductal adenocarcinoma^5,6^ is still recommended, as studies assessing adjuvant therapy for colloid carcinoma specifically have not been done.

## Conclusion

The cases presented in this article of colloid carcinoma represent a rare type of pancreatic cancer that has a significantly improved long-term survival compared to typical pancreatic ductal adenocarcinoma. Surgery is still considered the main treatment for this pathology, followed by adjuvant therapy as is currently recommended for ductal adenocarcinoma. It is important for clinicians to recognize the significantly improved survival for this diagnosis to be able to better counsel patients of their prognoses.

## Abbreviations Used

IPMN: intraductal papillary mucinous neoplasm
CT: computed tomography
EUS: endoscopic ultrasound
MRI: magnetic resonance imaging
PET: positron emission tomography
CA 19-9: carbohydrate antigen 19-9
